# Increased [^18^F]FMISO accumulation under hypoxia by multidrug-resistant protein 1 inhibitors

**DOI:** 10.1186/s13550-021-00752-3

**Published:** 2021-01-25

**Authors:** Yoichi Shimizu, Yukihiro Nakai, Hiroyuki Watanabe, Shimpei Iikuni, Masahiro Ono, Hideo Saji, Yuji Kuge, Tsuneo Saga, Yuji Nakamoto

**Affiliations:** 1 Department of Diagnostic Imaging and Nuclear Medicine, Graduate School of Medicine, Kyoto University, 54 Shogoinkawahara-cho, Sakyo-Ku, Kyoto, 606-8507 Japan; 2 Department of Patho-Functional Bioanalysis, Graduate School of Pharmaceutical Sciences, Kyoto University, Kyoto, 606-8501 Japan; 3 Central Institute of Isotope Science, Hokkaido University, Sapporo, 060-0815 Japan

**Keywords:** [^18^F]FMISO, Hypoxia, MRP1

## Abstract

**Background:**

[^18^F]Fluoromisonidazole ([^18^F]FMISO) is a PET imaging probe widely used for the detection of hypoxia. We previously reported that [^18^F]FMISO is metabolized to the glutathione conjugate of the reduced form in hypoxic cells. In addition, we found that the [^18^F]FMISO uptake level varied depending on the cellular glutathione conjugation and excretion ability such as enzyme activity of glutathione-S-transferase and expression levels of multidrug resistance-associated protein 1 (MRP1, an efflux transporter), in addition to the cellular hypoxic state. In this study, we evaluated whether MRP1 activity affected [^18^F]FMISO PET imaging.

**Methods:**

FaDu human pharyngeal squamous cell carcinoma cells were pretreated with MRP1 inhibitors (cyclosporine A, lapatinib, or MK-571) for 1 h, incubated with [^18^F]FMISO for 4 h under hypoxia, and their radioactivity was then measured. FaDu tumor-bearing mice were intravenously injected with [^18^F]FMISO, and PET/CT images were acquired at 4 h post-injection (1st PET scan). Two days later, the same mice were pretreated with MRP1 inhibitors (cyclosporine A, lapatinib, or MK-571) for 1 h, and PET/CT images were acquired (2nd PET scan).

**Results:**

FaDu cells pretreated with MRP1 inhibitors exhibited significantly higher radioactivity than those without inhibitor treatment (cyclosporine A: 6.91 ± 0.27, lapatinib: 10.03 ± 0.47, MK-571: 10.15 ± 0.44%dose/mg protein, *p* < 0.01). In the in vivo PET study, the SUV_mean_ ratio in tumors [calculated as after treatment (2nd PET scan)/before treatment of MRP1 inhibitors (1st PET scan)] of the mice treated with MRP1 inhibitors was significantly higher than those of control mice (cyclosporine A: 2.6 ± 0.7, lapatinib: 2.2 ± 0.7, MK-571: 2.2 ± 0.7, control: 1.2 ± 0.2, *p* < 0.05).

**Conclusion:**

In this study, we revealed that MRP1 inhibitors increase [^18^F]FMISO accumulation in hypoxic cells. This suggests that [^18^F]FMISO-PET imaging is affected by MRP1 inhibitors independent of the hypoxic state.

## Background

In solid tumor tissues, a low oxygen concentration region or hypoxic region is known to be related to cancer resistance toward radiotherapy and chemotherapy [1]. Thus, precise monitoring of hypoxic states in tumor tissue by positron emission tomography (PET) may provide useful information for determining optimal therapeutic strategies and individualized cancer treatment [2].

For a diagnosis of hypoxia by PET, many kinds of nitroimidazole-based agents have been developed [3]. Among them, [^18^F]fluoromisonidazole ([^18^F]FMISO) is widely used in both basic research and clinically [4]. We previously investigated the accumulation mechanism of [^18^F]FMISO using a combination of imaging mass spectrometry and radioisotope analysis and found that [^18^F]FMISO taken up by hypoxic cells was metabolized mainly to be the glutathione conjugate of reduced FMISO [5]. In addition, we performed a cellular uptake study of [^18^F]FMISO with several types of tumor cells whose glutathione conjugation and excretion ability, such as enzyme activity of glutathione-S-transferase and expression levels of multidrug resistance-associated protein 1 (MRP1), differed [6, 7]. In that study, the [^18^F]FMISO uptake level varied depending on the glutathione conjugation and excretion ability of cells, in addition to the cellular hypoxic state.

MRP1 is an ATP-binding cassette transporter that mediates the ATP-dependent export of glutathione conjugates out of cells [8]. Various kinds of agents clinically used for treatment have been reported to be able to block the transporter function of MRP1 [9]. In addition, MRP1 inhibitors have been reported to improve chemotherapy drug response in cancer [10]. Thus, we hypothesized that the agents blocking MRP1 activity affect the efflux of [^18^F]FMISO in hypoxic regions of tumors. In this study, we evaluated the accumulation levels of [^18^F]FMISO in hypoxic cells after pretreatment with compounds (cyclosporine A, lapatinib, and MK-571), which have been reported to inhibit the transporter activity of MPR1 [11–13], and performed in vivo PET imaging of [^18^F]FMISO before and after treatment with MPR1 blockers.

## Materials and methods

### Chemicals and reagents

All chemicals were commercially available and of the highest available purity. [^18^F]FMISO was synthesized as previously described [14].

### MRP1 activity assay

FaDu human head and neck cancer cells (American Type Culture Collection, Manassas, VA, USA) were maintained in Eagle’s Minimum Essential Medium (EMEM) (Sigma-Aldrich, St Louis, MO, USA) supplemented with 10% fetal bovine serum and penicillin (100 u/mL)-streptomycin (100 µg/mL) at 37 °C in a humidified atmosphere of 95% air and 5% CO_2_. MRP1 activity levels within the FaDu cells with MRP1 inhibitors were measured using the Multi-Drug Resistance Assay Kit (CAYMAN, Ann Arbor, MI, USA). In brief, FaDu cells were pretreated with EMEM containing cyclosporine A (0–100 µM), lapatinib (0–50 µM), or MK-571 (0–100 µM) for 1 h. Then, the cells were washed twice with phosphate-buffered saline (PBS), and the cells were treated with calcein AM, which is excreted out of cells by MRP1, and the fluorescence intensity of the cells was measured after additional incubation for 1 h. The samples were lysed with 1 N NaOH, and total protein concentrations were measured by the bicinchoninic acid (BCA) assay.

### Cellular uptake study

FaDu cells cultured in 6-well plates (1 × 10^6^ cells/2 mL EMEM) were preincubated for 18 h either under normoxic conditions at 37 °C in a humidified atmosphere containing 5% CO_2_ or under hypoxia at reduced oxygen levels (1% v/v) in a multigas incubator (APM–30D; ASTEC Co., Ltd., Fukuoka, Japan). Then, the cells were pretreated with cyclosporine A (100 µM), lapatinib (50 µM), or MK-571 (100 µM), or not pretreated (non-pretreated group) for 1 h under hypoxia. After the pretreatment, [^18^F]FMISO (5 MBq/2 mL EMEM) was added, and the cells were incubated under hypoxic or normoxic conditions (non-pretreated normoxia group). At 4 h post-incubation, the cells were washed three times with PBS and lysed with 1 N NaOH. The radioactivity (counts per minute, cpm) in the lysates was measured with a gamma counter (Wallac WIZARD 2470, PerkinElmer, Waltham, MA, USA), and the protein concentrations of the cell lysates were measured by the BCA assay. The radioactivity (cpm) of [^18^F]FMISO added to the cells was also measured with a gamma counter (Wallac WIZARD 2470). The cellular uptake level of [^18^F]FMISO is represented as “%dose/mg protein” as follows:

$$\% {\text{dose}}/{\text{mg protein}} = \frac{{100 \times [ {\text{radioactivity of cell lysate (cpm)}}]}}{{[ {\text{radioactivity of}}\,[18{\text{F}}]\,{\text{FMISO added to the cells (cpm)}}] \times [{\text{protein amount of cell lysate (mg)}})}}$$

### Tumor xenograft model

Five-week-old male BALB/c athymic nude mice supplied by Japan SLC, Inc. (Hamamatsu, Japan) were housed under a 12-h light/12-h dark cycle, with food and water supplied ad libitum. FaDu cells (5 × 10^6^ cells) suspended in 100 µL of PBS were injected subcutaneously into the right flank of each mouse. Further experiments were performed after a 2- or 3-week tumor growth period for the FaDu xenograft models. All animal manipulations were performed using sterile techniques.

### PET imaging study

[^18^F]FMISO (approximately 3.7 MBq/100 µL) was intravenously injected into FaDu-xenografted model mice. PET imaging was performed using a G8 small-animal PET/CT scanner (PerkinElmer). At 4 h after injection, the mice were anesthetized with 1.5–2.0% isoflurane, and then, static PET scans (scan time: 10 min) and CT scans (X-ray sources: 50 kVp, 200 µA) were performed. At 2 days after PET/CT scanning, the mice were intraperitoneally treated with a single dose of lapatinib (2.5 mg/1 mL 5% Tween80) (n = 5), MK-571 (2.0 mg/1 mL 5% Tween80) (n = 5), cyclosporine A (1.25 mg/1 mL 5% Tween80) (n = 6), or saline with 5% Tween80 (1 mL) (n = 6). At 1 h after the injection, [^18^F]FMISO (3.7 MBq/100 µL) was injected intravenously, and 4 h later, PET and CT scanning were performed using the same method as above. The PET data were reconstructed using a 3-dimensional maximum-likelihood expectation maximization (3D-MLEM) algorithm with CT-based attenuation correction. The acquired PET/CT images were analyzed using VivoQuant Software version 4.2 (inviCRO, Boston, MA, USA). A three-dimensional region of interest (ROI) was manually defined for the tumor in each mouse, and the [^18^F]FMISO accumulation levels in each tumor were quantified by calculating the mean standardized uptake value (SUV_mean_).

### Statistics

Data are presented as the mean ± S.E.M (for the in vitro study) or the mean ± S.D (for the in vivo study). Statistical analyses were performed with 2-way ANOVA following the Tukey–Kramer test. The statistical analyses were performed using JMP 14 software (SAS Institute Inc., Cary, NC, USA). A 2-tailed value of *p* < 0.05 was considered significant.

## Results

### In vitro MRP1 activity assay

The %intensity was calculated as the fluorescence intensity/mg protein of the cells pretreated with compounds (cyclosporine A, lapatinib, or MK-571) per the fluorescence intensity/mg protein of the non-treated cells. After pretreatment with cyclosporine A, lapatinib, or MK-571, the %intensity increased in a concentration-dependent manner (Additional file 1: Fig. S1). Based on these results, we used cyclosporine A (100 µM), lapatinib (50 µM), and MK-571 (100 µM) as MRP1 inhibitors in the subsequent in vitro study.

### In vitro FMISO uptake study

**Fig. 1:**
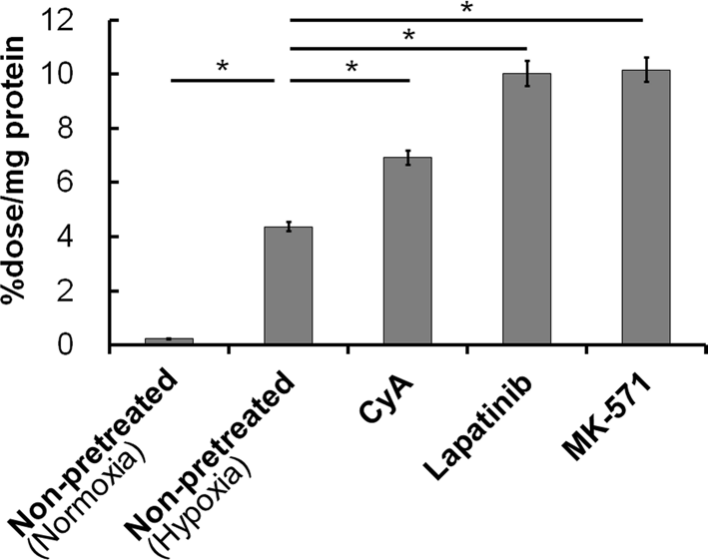
Uptake of [^18^F]FMISO at 4 h post-incubation by FaDu cells pretreated with cyclosporine A (CyA), lapatinib, or MK-571 under hypoxic (1% O_2_) or normoxic (only non-pretreated group) conditions. *p < 0.01

The cellular uptake of [^18^F]FMISO is expressed as the %dose/mg protein (Fig. 1). The cellular uptake level of [^18^F]FMISO under hypoxia was significantly higher than that under normoxia (hypoxia: 4.36 ± 0.17, normoxia: 0.22 ± 0.01 %dose/mg protein, *p* < 0.01). Furthermore, the cells pretreated with MRP1 inhibitors exhibited significantly higher radioactivity than those without inhibitor treatment (cyclosporine A: 6.91 ± 0.27, lapatinib: 10.03 ± 0.47, MK-571: 10.15 ± 0.44 %dose/mg protein, *p* < 0.01).

### PET/CT study

**Fig. 2:**
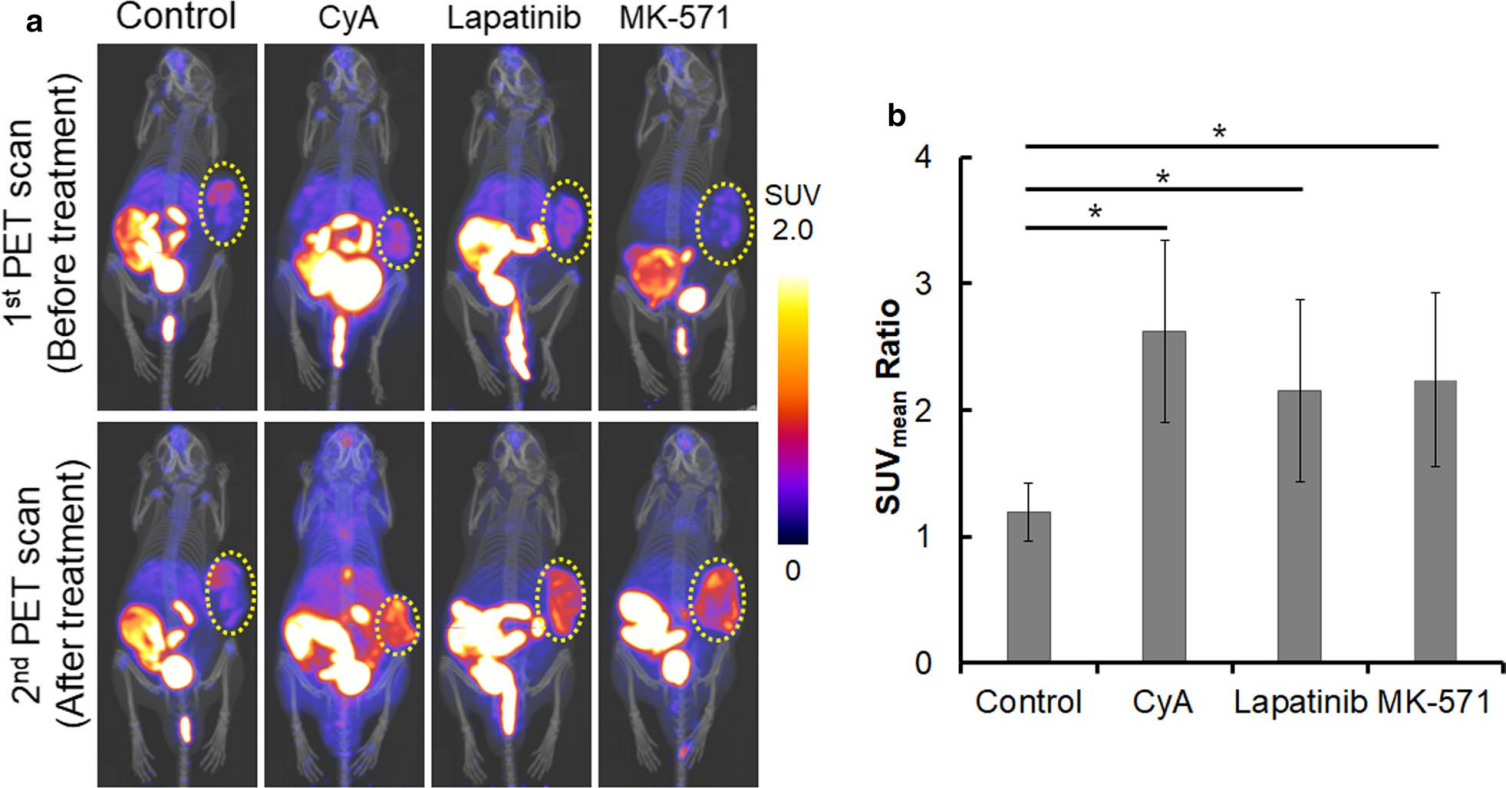
[^18^F]FMISO-PET imaging of FaDu tumor-xenografted mice pretreated with MRP1 inhibitors [cyclosporine A (CyA), lapatinib, or MK-571]. **a** Representative [^18^F]FMISO-PET/CT images of FaDu tumor-xenografted mice. The PET/CT images (1st PET scan) were acquired from FaDu tumor-xenografted mice at 4 h after i.v. administration of [^18^F]FMISO. Two days after the 1st PET scan, the mice were pretreated with CyA, lapatinib, MK-571, or 5%Tween80 (control), and then, the PET/CT images of [^18^F]FMISO (2nd PET scan) were acquired from the same mice in the 1st PET scan. The yellow circle indicates tumor tissues. **b** SUV_mean_ ratio of FaDu tumor tissues acquired from the PET images. The SUV_mean_ ratio was calculated as after treatment (2nd PET scan)/before treatment (1st PET scan). *p < 0.05

PET/CT images are shown as coronal maximum intensity projections (MIPs) (Fig. 2a). The SUV_mean_ ratio in tumors was calculated as after treatment (2nd PET scan)/before treatment with MRP1 inhibitors (1st PET scan). The SUV_mean_ ratios of mice treated with MRP1 inhibitors (cyclosporine A, lapatinib, or MK-571) were significantly higher than those of control mice (only 5% Tween80) (cyclosporine A: 2.6 ± 0.7, lapatinib: 2.2 ± 0.7, MK-571: 2.2 ± 0.7, control: 1.2 ± 0.2, *p* < 0.05) (Fig. 2b).

## Discussion

In this study, we focused on MRP1 as a key factor affecting the accumulation level of [^18^F]FMISO in hypoxic regions of tumors. MRP1 is an ATP-binding cassette transporter and is well known to mediate efflux of antineoplastic agents conjugated by glutathione, which confers multidrug resistance in a various kind of cancer [15]. In our previous study, we revealed that [^18^F]FMISO taken up in hypoxic tumor cells existed mainly as a reductive metabolite conjugated with glutathione, and the cells with higher mRNA expression of MRP1 showed lower [^18^F]FMISO accumulation level in the same hypoxic condition [6]. To consider that MRP1 mediates the efflux of agents conjugated with glutathione out of cells, we hypothesized that [^18^F]FMISO uptake in hypoxic cells might be correlated inversely with MRP1 activity, that is, the agents blocking MRP1 activity affect the efflux of [^18^F]FMISO in hypoxic regions of tumors. To verify this hypothesis, we planned to perform the in vitro and in vivo studies of [^18^F]FMISO under the pretreatment of MRP1 inhibitors.

We first confirmed that cyclosporine A, lapatinib, and MK-571 worked as MRP1 inhibitors (Additional file 1: Fig. S1) and found the optimal dose of those compounds for the following inhibition assay of cellular MRP1 activity. After that, we performed a cellular uptake study of [^18^F]FMISO after pretreatment with the selected compounds to reveal whether MRP1 inhibitors affect the [^18^F]FMISO uptake by tumor cells under hypoxic conditions. The accumulation levels of [^18^F]FMISO in FaDu cells cultured under hypoxia increased in an MRP-1 inhibitor concentration-dependent manner (Fig. 1), suggesting that MRP1 inhibitors increase the accumulation of [^18^F]FMISO in FaDu cells as we expected. Since [^18^F]FMISO accumulation is predicted to decrease by inhibition of glutathione S-transferase (GST) enzyme activity from our previous study [6], we also evaluated whether MRP1 inhibitors (cyclosporine A and lapatinib) affected GST activity. As a result, we found that those compounds inhibited the GST activity slightly (Additional file 1: Fig. S2B), which was opposite to the result of in vitro cellular uptake study of [^18^F]FMISO (Fig. 1). Thus, we supposed that the enhancement of [^18^F]FMISO accumulation observed in the in vitro cellular uptake study was derived from the MRP1 inhibition activities of the pretreated MRP1 inhibitors. It is also reported that treatment with a MRP1 inhibitor increased the reduced type of glutathione (GSH) and then suppressed the production of oxidative reactive oxygen species (ROS) in cells [16]. To consider that [^18^F]FMISO is supposed to accumulate in hypoxic cells via the reaction with GSH after reduction of its nitro group [5, 17], those conditions would be favorable for [^18^F]FMISO accumulation in hypoxic cells. Therefore, our results would support that [^18^F]FMISO accumulation in hypoxic cells depends not only on low oxygen level around the cells but also on the cellular MRP1 activity.

**Fig. 3:**
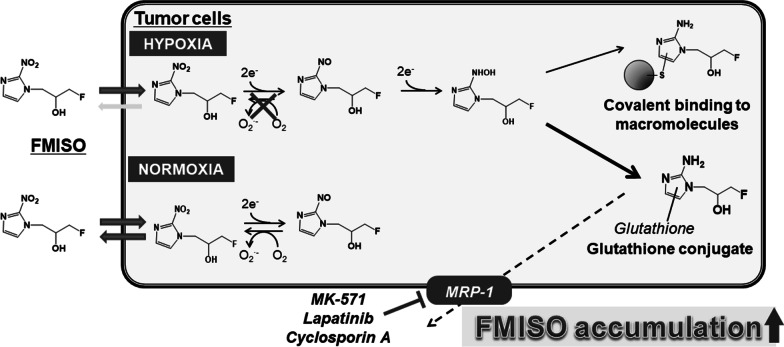
Proposed mechanism of increased [^18^F]FMISO accumulation under hypoxia by MRP1 inhibitors

In the PET imaging study, higher radioactivity in tumor tissues was observed after treatment with MRP1 inhibitors, and the SUV_mean_ ratios (2nd PET scan/1st PET scan) increased significantly by treatment with MRP1 inhibitors (Fig. 2). This suggests that [^18^F]FMISO accumulation in tumor tissues increased after treatment with agents able to block MRP1 transporter function (Fig. 3). To compare the intratumoral distribution of [^18^F]FMISO between 1st and 2nd PET scans, heterogeneous enhancement of the hypoxic region was observed in the 2nd PET scan. It remains unclear whether this phenomenon was due to the intratumoral MRP1 expression, since we could not evaluate directly the change of MRP1 expression and distribution in tumor tissues, which would be the only limitation of this study. Focusing on hypoxia itself, it is reported that hypoxia moved transiently within the tumor within a day [18]. Considering that we performed the 2nd PET scan at 2 days after the 1st PET, we supposed the different heterogeneous [^18^F]FMISO accumulation between 1st and 2nd PET scans was due to the movement of hypoxic region in tumor tissues.

In our previous study, we demonstrated that under the same hypoxic state, [^18^F]FMISO accumulated highly in cells with higher glutathione S-transferase (GST) enzyme activity and lower MRP1 expression [6]. In this study, we also evaluated whether the [^18^F]FMISO uptake was affected by GST inhibitors. We selected tannic acid as a GST inhibitor (Additional file 1: Fig. S2A). In the in vitro cellular uptake study of [^18^F]FMISO, the radioactivity of FaDu cells pretreated with tannic acid was significantly lower than that of non-pretreated cells (tannic acid group: 0.61 ± 0.06 vs. non-pretreated group: 3.82 ± 0.89 %dose/mg protein) (Additional file 1: Fig. S3). This result suggests that GST inhibitors can suppress the [^18^F]FMISO cellular uptake under hypoxic conditions. We also performed an in vivo biodistribution study of [^18^F]FMISO with tannic acid pretreatment; however, the radioactivity of tumor tissues from the mice pretreated with tannic acid was almost the same as that in tissues from the non-pretreated mice [tannic acid group: 1.50 ± 0.88 vs. non-pretreated group: 1.48 ± 0.59 %injected dose per tumor weight (%ID/g)] (Additional file 1: Fig. S4). In this study, we could not evaluate the GST activity in tumor tissues of mice with or without pretreatment of tannic acid. Therefore, it is also possible to consider that the dose of tannic acid was insufficient to block intratumoral GST activity, and thus, it remains unclear whether inhibition of GST activity really affects [^18^F]FMISO-PET imaging of tumor hypoxia. Although further detailed evaluation of the effects of GST inhibitors should be performed, our study suggests that the glutathione conjugation and excretion ability, especially the MRP1 activity of the cells, affect the [^18^F]FMISO accumulation in addition to the hypoxic state.

To date, MRP1 is focused on as a target biomolecule of cancer chemotherapy not only for the suppression of chemoresistance but also for the collateral sensitivity such as modulation of intratumoral oxidative stress [19]. Thus, various kinds of MRP1 inhibitors have been developed and reported [9]. Therefore, undergoing such chemotherapy, it should be paid attention to the enhancement of [^18^F]FMISO uptake in tumor tissues, which might cause overestimation of tumor hypoxia. In addition to [^18^F]FMISO we evaluated in this study, other nitroimidazole-based agents have also been reported to accumulate in hypoxic cells via the same mechanism as [^18^F]FMISO [20–22]. Furthermore, besides the tumor cells, we recently found that [^18^F]FMISO accumulation levels of macrophages in hypoxia were differently dependent on their phenotypes (polarized types) [23]. Therefore, the increased accumulation by MRP1 inhibitors should be considered when we use nitroimidazole-based PET imaging agents for diagnosing hypoxia not only in tumor tissues but also other pathological tissues.

In conclusion, we revealed that MRP1 inhibitors increase [^18^F]FMISO accumulation in hypoxic cells. This suggests that [^18^F]FMISO-PET imaging is affected by MRP1 inhibitors independent of the hypoxic state.

## Supplementary Information

**Additional file 1:** Materials and Methods, Figures of GST activity assay. Cellular uptake study. Animal experiments. Statistics. **Figure S1**. MRP1 activity of FaDu cells pretreated with cyclosporine A (A), lapatinib (B), or MK-571 (C). **Figure S2**. GST activity of FaDu cells pretreated with tannic acid (A), cyclosporine A, or lapatinib (B). **Figure S3**. Cellular uptake of [^18^F]FMISO at 4 h post-incubation with FaDu cells pretreated with tannic acid under hypoxic (1% O_2_) or normoxic (only non-pretreated group) conditions. **Figure S4**. The radioactivity (%ID/g) of tumor tissues of FaDu xenografted mice with or without tannic acid pretreatment (control) at 4 h after i. v. administration of [^18^F]FMISO.

## Data Availability

The datasets supporting the conclusions of this article are included within the article.

## Abbreviations

[^18^F]FMISO: [^18^F]Fluoromisonidazole
PET: Positron emission tomography
MRP1: Multidrug resistance-associated protein
GST: Glutathione-S-transferase
EMEM: Eagle’s Minimum Essential Medium
PBS: Phosphate-buffered saline
