# Supplementary material for: Increased [18F]FMISO accumulation under hypoxia by multidrug-resistant protein 1 inhibitors
Source: EJNMMI Res. 2021 Jan 25;11:9. doi: 10.1186/s13550-021-00752-3 (PMC7835267; doi:10.1186/s13550-021-00752-3)
Supplement: Supplementary file 1 — Additional file 1: Materials and Methods, Figures of GST activity assay. Cellular uptake study. Animal experiments. Statistics. Figure S1. MRP1 activity of FaDu cells pretreated with cyclosporine A (A), lapatinib (B), or MK-571 (C). Figure S2. GST activity of FaDu cells pretreated with tannic acid (A), cyclosporine A, or lapatinib (B). Figure S3. Cellular uptake of [18F]FMISO at 4 h post-incubation with FaDu cells pretreated with tannic acid under hypoxic (1% O2) or normoxic (only non-pretreated group) conditions. Figure S4. The radioactivity (%ID/g) of tumor tissues of FaDu xenografted mice with or without tannic acid pretreatment (control) at 4 h after i. v. administration of [18F]FMISO. [file 13550_2021_752_MOESM1_ESM.docx]

**Supplementary Materials and Methods**

*GST activity assay*

GST enzyme activity levels within the FaDu cell supernatants with the GST inhibitor were measured using the Glutathione S-Transferase Assay Kit (Sigma-Aldrich, St Louis, MO, USA). In brief, FaDu cells were incubated with EMEM containing tannic acid (0 - 100 μM), cyclosporine A (100 μM), or lapatinib (50 μM) for 1 h. After the incubation, the cells were lysed with 100 mM potassium phosphate (pH 7.0) containing 2 mM EDTA and then centrifuged (1,000×g, 15 min, 4°C). The supernatants were mixed with PBS, 200 mM GSH, and 100 mM 1-chloro-2,4-dinitrobenzene (CDNB). The reaction was measured in terms of an increase in the absorbance signal at 340 nm of the reaction product, i.e. the glutathione conjugate of CDNB, using the microplate reader. The rate of increase in absorption is directly proportional to the GST activity level in the sample. The samples were lysed with 1 N NaOH and total protein concentrations were measured by the BCA assay.

*Cellular uptake study*

FaDu cells cultured in 6-well plates (1 × 10^6^ cells/2 mL EMEM) were pre-incubated for 18 h either under normoxic conditions at 37°C in a humidified atmosphere containing 5% CO_2_ or under hypoxia at reduced oxygen levels (1% v/v) in a multi-gas incubator (APM–30D; ASTEC Co., Ltd., Fukuoka, Japan). Then, the cells were pretreated with tannic acid (100 μM) or not pretreated (non-pretreated group) for 1 h under hypoxia. After the pretreatment, [^18^F]FMISO (5 MBq/2 mL EMEM) was added and the cells were incubated under hypoxic conditions. At 4 h post-incubation, the cells were washed three times with PBS and lysed with 1 N NaOH. The radioactivity in the lysates was measured with a gamma counter (Wallac WIZARD 2470, PerkinElmer, Waltham, MA, USA) and the protein concentrations of the cell lysates were measured by the BCA assay.

*Animal experiments*

A single dose of tannic acid (2.0 mg/1 mL 5% Tween80) (n = 4) or saline with 5% Tween80 (1 mL) (control group, n = 5) was injected into the abdominal cavity of FaDu xenograft model mice. At 1 hour after the injection, [^18^F]FMISO (10 MBq/100 μL) was injected into the mice via the tail vein, and 4 h later, the mice were sacrificed. The tumor tissues were immediately excised and their radioactivity was measured with a gamma counter (Wallac WIZARD 2470).

*Statistics*

Data are presented as the mean ± S.E.M. (for the *in vitro* study) or the mean ± S.D. (for the *in vivo* study). Statistical analyses were performed with 2-way ANOVA following the Tukey–Kramer test (for the GST activity assay) or the Student’s t-test (for other studies). The statistical analyses were performed using JMP 14 software (SAS Institute Inc., Cary, NC, USA). A 2-tailed value of *p* < 0.05 was considered significant.

**Supplementary Figures**

**
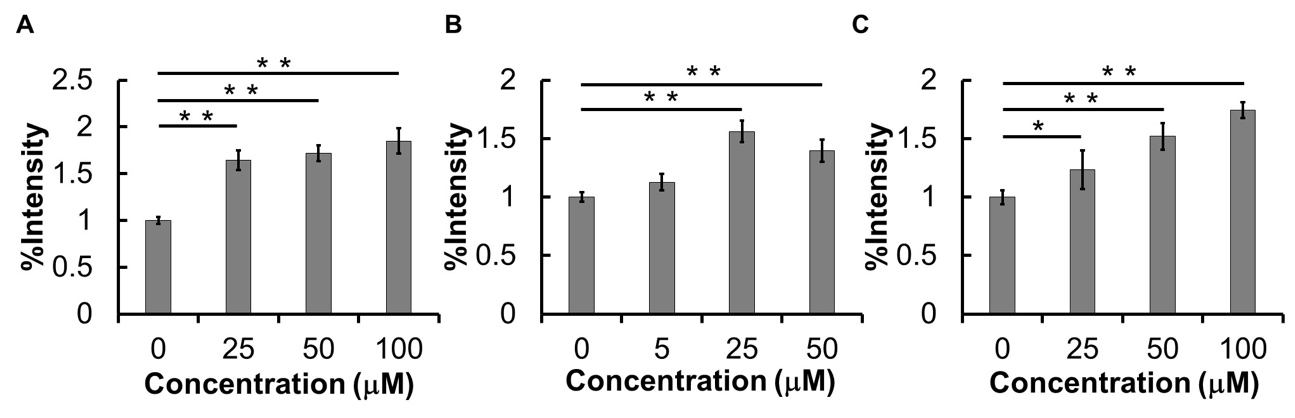
**

**Figure S1**

MRP1 activity of FaDu cells pretreated with cyclosporine A (A), lapatinib (B), or MK-571 (C). **p*<0.05, ***p*<0.01.


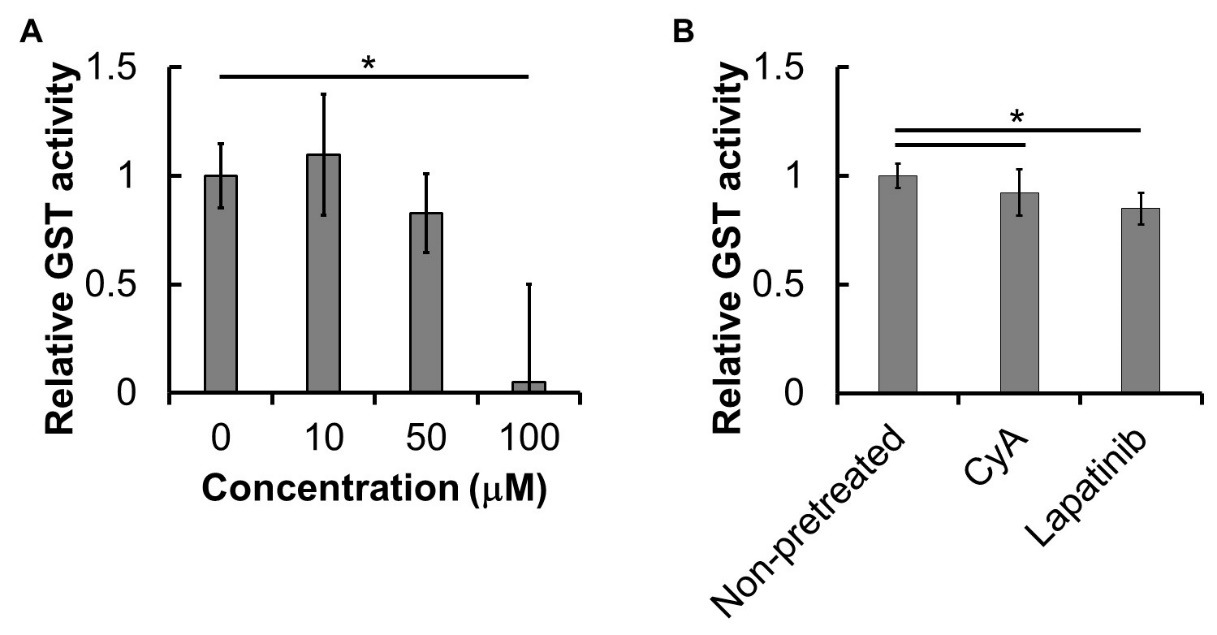


**Figure S2**

GST activity of FaDu cells pretreated with 0-100 μM tannic acid (A), 100 μM cyclosporine A (CyA), or 50 μM lapatinib (B). **p*<0.01.


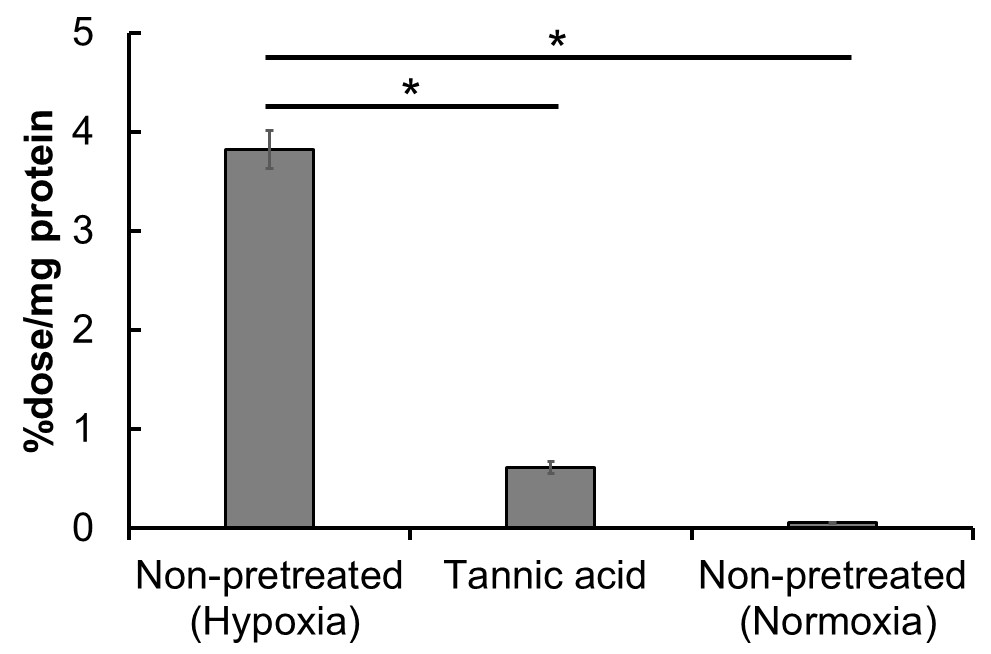


**Figure S3**

Cellular uptake of [^18^F]FMISO at 4 h post-incubation with FaDu cells pretreated with tannic acid under hypoxic (1% O_2_) or normoxic (only non-pretreated group) conditions. **p*<0.01.


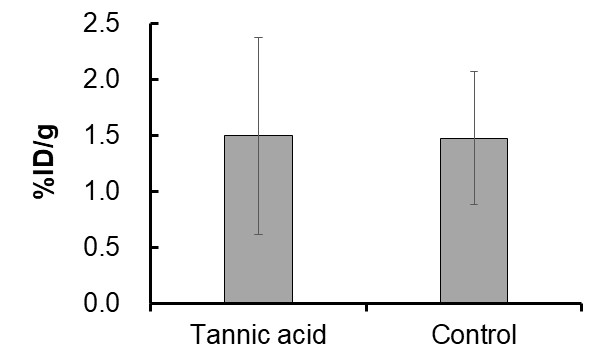


**Figure S4**

The radioactivity (%ID/g) of tumor tissues of FaDu xenografted mice with or without tannic acid pretreatment (control) at 4 h after i. v. administration of [^18^F]FMISO.
